# Point-of-care ultrasound (POCUS) practices in the helicopter emergency medical services in Europe: results of an online survey

**DOI:** 10.1186/s13049-021-00933-y

**Published:** 2021-08-26

**Authors:** Peter Hilbert-Carius, Manuel F. Struck, Marcus Rudolph, Jürgen Knapp, Leif Rognås, Jörn Adler, Cor Slagt, Lars Jacobsen, Henryk Pich, Michael D. Christian, Didier Dandrifosse, Fikri M. Abu-Zidan, POCUS in HEMS collaborators, Alistair Maddock, Anatolij Truhlar, Antonio Joaosagla, Daniel Nevin, Daniel Werner, Didier Moens, Diego Aylagas, Eimhea Quinn, Eva Smrzova, Frederic Pernot, Fredrik Helliksson, Geert Jan van Geffen, Gernot Aichinger, Jason van derde Velde, John Chatterjee, Jörg Seifert, Kirsti Strømmen Holm, Manfred Hirner, Marcel de Leeuw, Marcin Kowalski, Marton Radnai, Niko Rebling, Philipp Lakatos, Rein Ketelaars, Richard Lyon, Robert Galazkowski, Robert Gebei, Sebastian Daniel Tranca, Stephen Sollid, Uros Lampic, Urs Pietsch, Uwe Schweigkofler, Ville Voipio, Wolfgang Voelckel

**Affiliations:** 1 Department of Anesthesiology, Intensive Care, Emergency Medicine and Pain Therapy, BG Klinikum Bergmannstrost Halle gGmbH, and HEMS, Christoph 84” and “Christoph 85”, DRF-Luftrettung, Halle (Saale), Germany; 2 Department of Anesthesiology and Intensive Care Medicine, University Hospital Leipzig, Leipzig, and HEMS “Christoph 33”and “Christoph 71, Senftenberg, Germany; 3 DRF Stiftung Luftrettung Gemeinnützige AG, Filderstadt, Germany; 4 Department of Anaesthesiology and Pain Medicine, Inselspital, Bern University Hospital, University of Bern, Bern, Switzerland; 5 Danish Air Ambulance, Aarhus, Denmark; 6 Luxembourg Air Rescue A.S.B.L, Sandweiler, Luxembourg; 7 Department of Anesthesiology, Pain and Palliative Medicine and Helicopter Emergency Medical Service, Lifeliner 3” and “Lifeliner 5”, Radboudumc, Nijmegen, The Netherlands; 8 Sørlandet Hospital, Air Ambulance dpt, Arendal, Norway; 9 The Norwegian Air Ambulance Foundation, Arendal, Norway; 10 Department of Anesthesiology and Intensive Care Medicine, University Hospital Augsburg, Augsburg, Germany; 11 HEMS “Christoph 33”and “Christoph 71”, Senftenberg, Germany; 12 London’s Air Ambulance, Barth’s Health NHS Trust, London, UK; 13 Department of Surgery, College of Medicine and Health Science, UAE University, Al-Ain, United Arab Emirates

**Keywords:** Point-of-care ultrasound, Helicopter emergency medical service, Pre-hospital care, Emergency medicine, Survey

## Abstract

**Background:**

The extent to which Point-of-care of ultrasound (POCUS) is used in different European helicopter EMS (HEMS) is unknown. We aimed to study the availability, perception, and future aspects of POCUS in the European HEMS using an online survey.

**Method:**

A survey about the use of POCUS in HEMS was conducted by a multinational steering expert committee and was carried out from November 30, 2020 to December 30, 2020 via an online web portal. Invitations for participation were sent via email to the medical directors of the European HEMS organizations including two reminding notes.

**Results:**

During the study period, 69 participants from 25 countries and 41 different HEMS providers took part in the survey. 96% (n = 66) completed the survey. POCUS was available in 75% (56% always when needed and 19% occasionally) of the responding HEMS organizations. 17% were planning to establish POCUS in the near future. Responders who provided POCUS used it in approximately 15% of the patients. Participants thought that POCUS is important in both trauma and non-trauma-patients (73%, n = 46). The extended focused assessment sonography for trauma (eFAST) protocol (77%) was the most common protocol used. A POCUS credentialing process including documented examinations was requested in less than one third of the HEMS organizations.

**Conclusions:**

The majority of the HEMS organizations in Europe are able to provide different POCUS protocols in their services. The most used POCUS protocols were eFAST, FATE and RUSH. Despite the enthusiasm for POCUS, comprehensive training and clear credentialing processes are not available in about two thirds of the European HEMS organizations. Due to several limitations of this survey further studies are needed to evaluate POCUS in HEMS.

**Supplementary Information:**

The online version contains supplementary material available at 10.1186/s13049-021-00933-y.

## Background

Point-of-care of care ultrasound (POCUS) is a bedside, safe, diagnostic tool that can be repeatedly performed in sick patients to get useful information [1, 2]. It has been used more than thirty years ago in acute care settings [3]. The small size, light weight portability, improved quality of imaging, ability to store images, and the relatively low cost are clear advantages of the hand-held portable ultrasound machine [4]. These advantages make POCUS useful in many acute point-of-care settings including pre-hospital resuscitation, emergency departments, intensive care units, and operation theatres [1, 5]. POCUS performed in the pre-hospital and mass casualty incidents may affect the clinical decisions, notifications, transport modes, and hospital destination [4]. Pre-hospital POCUS was established two decades ago in various pre-hospital emergency medical services (EMS) in Europe, Australia, and North America [6, 7]. It was available in 9% of the French EMS units [8] and in 4.1% in the USA and Canada [9]. Furthermore, 21% of EMS services in the USA and Canada considered implementing it [9]. Pre-hospital POCUS is not widely used possibly due to limited availability and lack of strong evidence of its clinical value [10–12]. The extent to which POCUS is used in different European helicopter EMS (HEMS) is unknown. There exist no data on applied POCUS protocols, its training and credentialing methods, and the opinions of health care providers in the HEMS on its value. We aimed to study the availability, perception, and future aspects of POCUS in the European HEMS using an online survey that needs less than ten minutes to answer.

## Methods

A multinational steering expert committee of 12 experts from 7 countries developed the questionnaire about the use of POCUS in HEMS. Prerequisite for the questionnaire was the ability to answer all possible questions within 10 min and to include the availability, perception, and future aspects of POCUS in the European HEMS. After the agreement between of the experts about the basic areas to be addressed in the questionnaire, the first draft of the questionnaire was written by two of the authors (PH-C and FMA-Z), it was sent for other international experts for their input and modified accordingly. The first and second drafts of the questionnaire were edited via email while the third draft was edited online after sharing it. After approval from all experts, the survey was made available online. This implies that we depended mainly on surface validity for validation while content validity depended mainly on the experts’ experience in this area including one of the international experts who has more than 32 years’ experience in POCUS training and research including educational and qualitative research (FMA-Z). We did not pilot the questionnaire for linguistic clarity because it was reviewed by 12 experts who stemmed from 7 countries of different languages which assured that the questionnaire was clear. The ten-minutes survey consisted of 24 questions regarding demographics, availability, present and future use of POCUS in HEMS, importance of POCUS in different conditions, used POCUS protocols, and if there were any necessary credentialing POCUS processes for medical providers (Additional file 1: Table S1). The questionnaire was developed to determine the POCUS availability, used protocols and the prerequisites for its use by the medical staff. The survey was provided online via the web portal SurveyMonkey®. To ensure that every participant could only answer the survey once, the IP-address was recorded, whereas all data were analyzed anonymously. The invitation link and the QR-code for the survey was sent via email to the medical directors of 45 European HEMS organizations and Search and Rescue (SAR) bases of 28 countries across Europe with known HEMS use and a second and third reminding note was sent to non-respondents. The survey was online available from November 30 to December 30, 2020 and it was possible to answer it with any mobile device (smartphone, tablet) or PC.

Descriptive analysis was done using the analysis tools provided by SurveyMonkey® and the statistic software GraphPad Prism 9.0 (GraphPad Software, San Diego, CA, USA). Data were presented as median (range) and mean (SD) for ordinal and continuous data, and number (%) for categorical data. If data were missing, valid percentages were calculated from the available data.

The study is in line with the current European general data protection regulation (GDPR).

## Results

### General data

During the study period, 69 participants from 25 countries (89% of the invited 28 countries) and 41 different HEMS organizations (85% of the invited 45 HEMS organizations) took part in the survey. The survey was completed by 96% (n = 66 of 69) of the participants. Most of the participants 95.5% (n = 65 of 69) were males, between 41 and 50 years old, and had a leading position within their HEMS organization (71%, n = 49 of 69). Almost all HEMS programs (97.5%, n = 40 of 41) were physician staffed, in which the physician was joined by a paramedic in 65% (n = 26 of 40) or a flight nurse in 20% (n = 8 of 40) (Table 1). An Infirmier Siamu (Infirmier—French term for a nurse; Siamu—abbreviation for the French term “Soins Intensifs et Aide Medicale Urgente”; intensive care and urgent medical aid) a nurse that combines clinical intensive care medicine and preclinical emergency medicine, as well as HEMS-TC competency, were part of the medical team in 7.5% (n = 3 of 40), and a paramedic or flight nurse in 2.5% (n = 1 of 40) respectively (missing data were in 5%, n = 2). The non-physician staffed HEMS was paramedic only service.

**Table 1:** Overview of countries, HEMS-organizations (anonymous) and POCUS of the survey participants

Country	No. of responders	No. of providers that have answered	HEMS-organizations*	Physician staffed	With Doctor in cabin	POCUS provided
1) Austria	2	2	1	Yes	Paramedic	Yes
			2	Yes	Paramedic	Yes
2) Belgium	1	1	3	Yes	Infirmier Siamu	Yes
3) Czech	9	5	4	Yes	Paramedic	No
Republic			4	Yes	Paramedic	Yes
			4	Yes	Paramedic	No
			5	Yes	Paramedic	No
			4	Yes	Paramedic	No
			6	Yes	Flight nurse	Occasionally
			4	Yes	Paramedic	Yes
			7	Yes	Paramedic	No
			8	Yes	Paramedic	No
4) Denmark	1	1	9	Yes	Paramedic	Yes
5) Finland	2	1	10	Yes	Paramedic	Yes
6) France	2	2	11	Yes	Flight nurse	No
			12	Yes	Flight nurse	Occasionally
7) Germany	13	4	13	Yes	Paramedic	Yes
			14	Yes	Paramedic	Yes
			15	Yes	Paramedic	Yes
			16	Yes	Paramedic	Yes
8) Greece	1	1	17	yes	Flight nurse	Occasionally
9) Hungary	1	1	18	Yes	Paramedic	Yes
10) Ireland (Republic)	1	1	19	No		No
11) Israel	2	1	20	Yes	Paramedic	Occasionally
12) Italy	1	1	21	Yes	Flight nurse	Yes
13) Liechtenstein	1	1	22	Yes	Paramedic	No
14) Luxembourg	1	1	23	Yes	Infirmier Siamu	Yes
15) Netherland	6	1	24	Yes	Paramedic	Yes
16) Norway	5	1	25	Yes	Paramedic	Yes
17) Poland	1	1	26	Yes	Paramedic	Yes
18) Portugal	1	1	27	Yes	Infirmier Siamu	No
19) Romania	1	1	28	Yes	n.a	Occasionally
20) Russia	1	1	29	Yes	Paramedic	Occasionally
21) Slovenia	1	1	30	Yes	n.a	Yes
22) Spain	2	2	31	Yes	Flight nurse	Occasionally
			32	Yes	Flight nurse	No
23) Sweden	3	1	33	Yes	Flight nurse	Yes
24) Switzerland	5	3	34	Yes	Paramedic	No
			35	Yes	Paramedic	No
			36	Yes	Paramedic	Yes
25) United Kingdom	5	5	37	Yes	Paramedic	Yes
			38	Yes	Paramedic	No
			39	Yes	Paramedic	Yes
			40	Yes	Paramedic	Yes
			41	Yes	Paramedic	Yes

n.a.—no answer; *Number of the HEMS. is the anonymous unique identifier of the organisation to keep the data anonymous

### POCUS and HEMS organizations

Unrestricted availability of POCUS was given in 56% (n = 23 of 41) of the HEMS organizations (standardized equipment at all related HEMS bases), occasionally possible in 19.5% (n = 8 of 41), and not possible in 24.5% (n = 10 of 41) (Table 1). The time since POCUS had been established in the different HEMS organizations ranged from less than one year up to 20 years. Of the HEMS organizations not yet providing POCUS, 70% (n = 7 of 10) stated planning to integrate it in the future within a median (range) time of 2 (1–4) years. Responders of the HEMS providers in which POCUS was available estimated that POCUS had been used in a median (range) percentage of 15% (0.8–37.5) of treated patients (Table 2).

**Table 2:** Time since POCUS is provided or will be provided and frequents of use

Question	Number	Median (range)	Mean (SD)
For how many years have your HEMS been providing POCUS?	51	6 (0.5–20)	6.54 (4.4)
In how many years does your HEMS organization plan to integrate POCUS in the patients care in the future?	11	2 (1–4)	2.81 (1.1)
How often has POCUS being used in the last 1000 patients of your HEMS organization?	40	150 (8–375)	146 (100)
		15% (0.8–37.5)	14.6% (10)

Regarding the credentialing process for using POCUS in the different HEMS organizations providing POCUS, only 35% (n = 11 of 31) has an established credentialing process. If a credentialing process was established, a POCUS-course led by an expert was requested in 9 HEMS, an additional didactic teaching of an average of 6.5 h and hands-on training of an average of 5.5 h were requested in four HEMS. In two of the four mentioned HEMS organizations, documented POCUS cases were needed before using POCUS in HEMS. In two HEMS organizations, own didactic teaching and hands-on training were requested. Generally, comprehensive training and credentialing activities are scarce in the European HEMS organizations.

### Participants’ opinion

Table 3 summarizes the results of the importance of POCUS in general, in different areas and different patient conditions. Most participants think that POCUS is important in both trauma and non-trauma patients (73%, n = 46 of 63), whereas 19% (n = 12 of 63) think that POCUS is more important in trauma patients, while 8% (n = 5 of 63) think that it is important in non-trauma patients. Standard examination protocols are being used by the majority of participants 63% (n = 38 of 60), whereas 32% (n = 19 of 60) do not use such protocols and 5% (n = 3 of 60) were not sure. The (e)FAST protocol is the most used protocol (77%). The findings of POCUS were recorded in a reliable way (video clip or electronic database) in less than 30%, and mainly put down in writing on the mission protocol (Table 4).

**Table 3:** Importance of POCUS in general, in different areas and different patient conditions

	Number	Median (range)	Mean ± SD
**How important is POCUS for your HEMS organization in daily HEMS practice?**			
(1 not important at all, 10 extremely important)?			
	61	7 (1–10)	6.72 (2.19)
**What are the areas mainly investigated with POCUS in your HEMS and how important are they?**			
(1 not important, 2 possible importance, 3 important, 4 very important, 5 of utmost importance)			
Airway	55	2 (1–5)	2.04 (1.05)
Chest	60	4 (1–5)	3.85 (0.92)
Regional anesthesia	56	2 (1–4)	1.89 (0.89)
Abdomen	60	4 (1–5)	3.63 (0.92)
Echocardiography	58	4 (1–5)	3.69 (1.06)
Vascular (Aortic aneurysm)	58	3 (1–5)	2.98 (1.03)
**What are the clinical conditions in which POCUS is important?**			
(1–I disagree, 2–I am not sure, 3–I agree			
Traumatic shock	60	3 (1–3)	2.92 (0.33)
Non traumatic shock	59	3 (1–3)	2.78 (0.5)
Acute abdomen	59	3 (1–3)	2.49 (0.78)
Dyspnea	60	3 (1–3)	2.77 (0.5)
CPR	60	3 (1–3)	2.68 (0.57)

**Table 4:** Used POCUS protocols and mode of recording of the findings

POCUS protocol	Number	%
**If you use standard protocols**–**What protocols are used?**		
(p)FAST (pre-hospital focused assessment sonography for trauma)	9	23
(e)FAST (extended focused assessment sonography for trauma)	30	77
FATE (focus assessed transthoracic echo)	14	36
RUSH (rapid ultrasound in shock)	10	26
Others (see below)	7	18
Not specified	5	12.5
FEEL (focused echocardiography in emergency life support)	1	2.5
Lung-US for COVID-19	1	2.5
**How are the POCUS findings recorded in your HEMS?**		
Mission protocol / Patient Record Form	33	57
Video clip	8	14
Electronic data base	8	14
Not recorded, if not relevant	11	19
Not recorded at all	16	28

### POCUS devices

The most commonly used portable ultrasonography devices were, GE healthcare V-scan in 40% (n = 21), FUJIFILM Sonosite iviz in 36% (n = 19), Philips healthcare Lumify and Butterfly Network iQ in 6% (n = 3) respectively. Some HEMS organizations use more than one POCUS device manufacturer. Most of the participants (71%, n = 39) were pleased with the devices used.

## Discussion

Our study indicates that more than two-thirds of the European HEMS organizations provide POCUS in their helicopters and that a considerable number is planning to establish it soon. HEMS providers appreciate the increased need for POCUS integration in pre-hospital care. To our knowledge, this is the first survey regarding the pre-hospital use of POCUS in HEMS organizations across Europe.

Data suggest that POCUS is feasible and useful in HEMS. Nevertheless, the evidence regarding improving direct patient outcome is weak which needs properly designed prospective studies [10, 11, 13–18]. There are different POCUS protocols that can be used in the pre-hospital setting which include extended (e)FAST to search for intraperitoneal fluid, peri-cardiac fluid, haemothorax and pneumothorax, [19, 20], Rapid Ultrasound for Shock (RUSH) to define the cause of the shock, and Focused Assessment Transthoracic Echocardigraphy (FATE) or Focused Echocardiography in Emergency Life support (FEEL) to quickly evaluate the cardiac function [21–25]. Our results show, that (e)FAST is the most used protocol in HEMS. Independent of the used protocols whether (e)FAST, RUSH, FATE, FEEL or others, we think that it is important to carry out POCUS in patients in critical conditions or shock to find or exclude free fluid in the abdomen, in the thorax or in the pericardium, to detect or exclude pneumothorax, to find causes of shock and to exclude or confirm reversal causes of cardiac arrest. In this context POCUS is a physiological study, an on spot clinical decision tool, a clinical examination extension, a unique and expanding, safe and repeatable tool [1, 2].

With advancements in technology and training, the use of POCUS extended to more indications like diagnosis of eye injuries and bone fractures [26, 27]. POCUS training should be tailored towards the specific needs of the HEMS staff. The operators should be familiar with their own ultrasound machines and should be particularly knowledgeable of the sonographic artefacts that can mislead them [1, 28]. On the other hand, if the operators are familiar with their ultrasound machines they are able to make use of the record function of modern machines to record images or loops of the findings. As shown in Table 4, only minority of participants of this survey made use of the “record function” of their ultrasound machines. More than one quarter does not record the findings at all and more than 50% outline the findings in the mission protocol. Only 12% of the participants are doing both, recording as video and in the mission protocol (data not shown in Table 4). There is much potential for further improvement regarding this issue. This is very important for medicolegal issues, credentialing, closing the learning loop by reviewing the video clips, and using the clips for training and research so as to refine and advance the use of POCUS.

The participants thought that POCUS examinations of the chest, abdomen and heart are very important, vascular access are important, while POCUS for airway management and regional anesthesia is less important, (see Table 3). It is of interest to note that the needed POCUS skills for airway management and interventions are more advanced. Currently less than one-third of the participating HEMS organizations seems to have a credentialing process for using POCUS. The other two-third assumed that the HEMS crews can perform POCUS. Training must be standardized to maximize the benefit of POCUS. European HEMS organizations should agree on common POCUS curriculum with an accepted standard that suits their needs. Competency is a key factor in successful clinical applications [1, 29]. Using a Delphi methodology, Micheller et al. defined a total of five modalities (cardiac, thoracic, FAST, aorta, and procedural), with 32 measured competencies and 72 sub competencies [30]. Consecutive quality assurance and governance is probably more challenging, as POCUS findings are interpreted in a dynamic clinical context. The availability and operator acceptance of the POCUS equipment seem to be less of a challenge, at least in Europe.

Besides the more frequent use of POCUS compared with North America, the survey underlines that HEMS in Europe is mainly physician staffed which can explain the frequent use of POCUS [9, 29]. Some participants stated that POCUS is used in more than 30% of their patients indicating proper training in a wide range of applications.

### Limitations

The represented study has some limitations which we would like to highlight. First, it was a voluntary online survey that carries the risk of selection bias of participants who encourage the use of POCUS. This may overestimate the value of POCUS. Second, respondents were heterogeneous, from different levels, with unequal numbers from diferent organizations. Majority were leaders in their HEMS organization, with the risk of reporting results that are preferred by them and may be different from those who use it. We decided to analyse as many answers as possible because some HEMS providers do not provide uniform POCUS approaches. Not all helicopters are equally equipped (e.g. general availability of an ultrasound machine or type of ultrasound machine), even if they are operated by the same HEMS provider. Furthermore, some points of the questionnaire were about personal opinions of the participants, which are not identical. Third, we did not get the response of all invited HEMS organizations and we are unable to make sure, that all HEMS in Europa have been reached due to constant changes in the European HEMS scenery. This carries the risk of selection bias. The survey was asked in a limited period of 30 days possibly explaining the small sample size. Fourth, female responders were few with the majority being males. Fifth, no information regarding the time required to carry out POCUS and if there were any time limiting rules when carrying out POCUS were included in the survey. Sixth, we have to acknowledge that the current study is not a hypothesis testing study trying to answer a specific research question but aimed at collecting general data on the current status of POCUS use in Europe which will help us to define more hypothesis generating questions in the future. Accoridngly, specific details on each application (like the use of local anesthesia) are missing. Finally, some of the participating countries and HEMS organizations were over represented. This was taken into consideration when reporting availability of POCUS in the organizations but could have skewed the opinion data.

## Conclusions

Our study has shown that most of the HEMS organizations in Europe are able to provide different POCUS protocols in their services. The most used POCUS protocols were (e)FAST, FATE and RUSH. Despite the enthusiasm for POCUS, comprehensive training and clear credentialing processes are not available in about two thirds of the European HEMS organizations. The survey has several limitations which should be considered when interpreting the results. Due to these limitations further studies are needed to evaluate POCUS in HEMS.

## Supplementary Information

**Additional file 1.** Questionnaire as it was provided on the web page.

## Data Availability

The dataset generated and analysed during the current study are not publicly available due the ownership of the different air ambulance providers but are available from the corresponding author on reasonable request.

## Abbreviations

COVID: Corona virus infection disease
CPR: Cardio pulmonary resuscitation
EMS: Emergency medical service
FAST: Focused assessment sonography for trauma
FATE: Focused assessment transthoracic echocardiography
FEEL: Focused echocardiography in emergency life support
HEMS: Helicopter emergency medical service
n.a.: No answer
POCUS: Point-of-care ultrasound
RUSH: Rapid ultrasound in shock
SD: Standard deviation
SIAMU: Soins Intensifs et Aide Medicale Urgente “intensive care and urgent medical aid”
UK: United Kingdom

## References

[1] Abu-Zidan FM (2012). Point-of-care ultrasound in critically ill patients: Where do we stand?. J Emerg Trauma Shock.

[2] Khan MAB, Abu-Zidan FM (2020). Point-of-care ultrasound for the acute abdomen in the primary health care. Turk J Emerg Med.

[3] Abu-Zidan FM, Zayat I, Sheikh M, Mousa I, Behbehani A (1996). Role of ultrasonography in blunt abdominal trauma: a prospective study. Eur J Surg.

[4] Dittrich K, Abu-Zidan FM (2004). Role of ultrasound in mass-casualty situations. Int J Disaster Med.

[5] Abu-Zidan FM (2016). Optimizing the value of measuring inferior vena cava diameter in shocked patients. World J Crit Care Med.

[6] Ketelaars R, Reijnders G, van Geffen GJ, Scheffer GJ, Hoogerwerf N (2018). ABCDE of prehospital ultrasonography: a narrative review. Crit Ultrasound J.

[7] O'Dochartaigh D, Douma M (2015). Prehospital ultrasound of the abdomen and thorax changes trauma patient management: a systematic review. Injury.

[8] Bobbia X, Hansel N, Muller L, Claret PG, Moreau A, Genre Grandpierre R (2014). Availability and practice of bedside ultrasonography in emergency rooms and prehospital setting: a French survey. Ann Fr Anesth Reanim.

[9] Taylor J, McLaughlin K, McRae A, Lang E, Anton A (2014). Use of prehospital ultrasound in North America: a survey of emergency medical services medical directors. BMC Emerg Med.

[10] Portable Ultrasound Devices in the Pre-Hospital Setting: A Review of Clinical and Cost-Effectiveness and Guidelines. CADTH Rapid Response Reports. Ottawa (ON)2015. 26985544

[11] Stengel D, Rademacher G, Ekkernkamp A, Guthoff C, Mutze S. Emergency ultrasound-based algorithms for diagnosing blunt abdominal trauma. Cochrane Database Syst Rev. 2015(9):CD004446. 10.1002/14651858.CD004446.pub4 PMC6464800 26368505

[12] Botker MT, Jacobsen L, Rudolph SS, Knudsen L (2018). The role of point of care ultrasound in prehospital critical care: a systematic review. Scand J Trauma Resusc Emerg Med.

[13] Ultrasound in the Air Medical Environment (2018). Air Med J.

[14] Ketelaars R, Beekers C, Van Geffen GJ, Scheffer GJ, Hoogerwerf N (2018). Prehospital echocardiography during resuscitation impacts treatment in a physician-staffed helicopter emergency medical service: an observational study. Prehosp Emerg Care.

[15] Ketelaars R, Holtslag JJM, Hoogerwerf N (2019). Abdominal prehospital ultrasound impacts treatment decisions in a Dutch Helicopter Emergency Medical Service. Eur J Emerg Med.

[16] Ketelaars R, Hoogerwerf N, Scheffer GJ (2013). Prehospital chest ultrasound by a dutch helicopter emergency medical service. J Emerg Med.

[17] Mason R, Latimer A, Vrablik M, Utarnachitt R (2019). Teaching flight nurses ultrasonographic evaluation of esophageal intubation and pneumothorax. Air Med J.

[18] O'Dochartaigh D, Douma M, Alexiu C, Ryan S, MacKenzie M (2017). Utilization criteria for prehospital ultrasound in a canadian critical care helicopter emergency medical service: determining who might benefit. Prehosp Disaster Med.

[19] Maximus S, Figueroa C, Whealon M, Pham J, Kuncir E, Barrios C (2018). eFAST for pneumothorax: real-life application in an urban level 1 center by trauma team members. Am Surg.

[20] Mohammad A, Hefny AF, Abu-Zidan FM (2014). Focused assessment sonography for trauma (FAST) training: a systematic review. World J Surg.

[21] Gonzalez JM, Ortega J, Crenshaw N, de Tantillo L (2020). Rapid ultrasound for shock and hypotension: a clinical update for the advanced practice provider: part 2. Adv Emerg Nurs J.

[22] Gonzalez JM, Ortega J, Crenshaw N, de Tantillo L (2020). Rapid ultrasound for shock and hypotension: a clinical update for the advanced practice provider: part 1. Adv Emerg Nurs J.

[23] Kodaka M (2017). Cardiac point-of-care using ultrasound. Masui.

[24] Ozen C, Salcin E, Akoglu H, Onur O, Denizbasi A (2016). Assessment of ventricular wall motion with focused echocardiography during cardiac arrest to predict survival. Turk J Emerg Med.

[25] Scharonow M, Weilbach C (2018). Prehospital point-of-care emergency ultrasound: a cohort study. Scand J Trauma Resusc Emerg Med.

[26] Abu-Zidan FM (2017). Ultrasound diagnosis of fractures in mass casualty incidents. World J Orthop.

[27] Abu-Zidan FM, Balac K, Bhatia CA (2016). Surgeon-performed point-of-care ultrasound in severe eye trauma: report of two cases. World J Clin Cases.

[28] Abu-Zidan FM, Hefny AF, Corr P (2011). Clinical ultrasound physics. J Emerg Trauma Shock.

[29] Amaral CB, Ralston DC, Becker TK (2020). Prehospital point-of-care ultrasound: a transformative technology. SAGE Open Med.

[30] Micheller D, Peterson WJ, Cover M, Smith G, Chapman M, Theyyunni N (2019). Defining a theory-driven ultrasound curriculum for prehospital providers. Air Med J.

