# Supplementary material for: Point-of-care ultrasound (POCUS) practices in the helicopter emergency medical services in Europe: results of an online survey
Source: Scand J Trauma Resusc Emerg Med. 2021 Aug 26;29:124. doi: 10.1186/s13049-021-00933-y (PMC8390051; doi:10.1186/s13049-021-00933-y)
Supplement: Supplementary file 1 — Additional file 1. Questionnaire as it was provided on the web page. [file 13049_2021_933_MOESM1_ESM.pdf]

EXIT

Point of care ultrasound (POCUS) in European Helicopter Emergency Medical Services.

1. General Informtion

1. How old are you?

- ◯ 25-30
- ◯ 31-40
- ◯ 41-50
- ◯ 51-60
- ◯ > 60

2. What is your gender?

- ◯ female
- ◯ male

3. In which country is the HEMS you are working with based?

4. What HEMS organization / provider you are working with?

5. Do you have a leading position (medical superadvisor, medical leader of a HEMS base, chief instructor or training manager) within your HEMS organisation?

- ◯ Yes
- ◯ No

6. Is your HEMS physician staffed?

- ◯ Yes
- ◯ No

Next

Powered by
SurveyMonkey®
See how easy it is to create a survey .

Privacy & Cookie Polic y

POCUS in European HEMS

Point of care ultrasound (POCUS) in European Helicopter Emergency Medical Services.

2. Staff and POCUS

7. If the anwser of the previous question was **NO** - Is your HEMS staffed with a

- ☐ Pramedic
- ☐ Fligth Nurse
- ☐ Infirmier Siamu (nurse that combines clinical and preclinical medicine)

8. If the answer to question 6 was **YES -** What other profession is part of the HEMS medical team?"

- ◯ Paramedic
- ◯ Fligth Nurse
- ◯ Infirmier Siamu (nurse that combines clinical and preclinical medicine)

9. Does your HEMS organization provide POCUS?

- ◯ Yes, always when needed.
- ◯ Yes, but occasionally (only in some type of aircraft, or only on some bases ….)
- ◯ No

10. If the answer of the previous question was **YES** - For how many years have your HEMS been providing POCUS?

11. If the answer of question 8 was **NO** - Does your HEMS organization plan to integrate POCUS in the patients care in the future?

- Yes
- No
- Not sure

12. If the answer of the previous question was **YES** - When will you probably start to provide POCUS?

- Less than 1 year
- Within 2 years
- Within 3 years
- > 3 years

Prev Next

Powered by
SurveyMonkey®
See how easy it is to create a survey.

POCUS in European HEMS

Point of care ultrasound (POCUS) in European Helicopter Emergency Medical Services.

3. How important is POCUS?

13. How important is POCUS for your HEMS organization in daily HEMS practice (1-10, 1 not important at all, 10 extremely important)?

14. In what kind of patients do you think POCUS is more important?

- Trauma
- Non trauma
- Both

15. What are the areas mainly investigated with POCUS in your HEMS and how important are they? (Just give answers for areas that are investigated in your HEMS.)

	not important at all	possible important	important	very imortant	of utmost importance
Airway ultrasound					
Chest					
Regional anesthesia					
Abdomen					
Echocardiography					
Vascular (Aortic aneurysm)					

	not important at all	possible important	important	very imortant	of utmost importance
Others	○	○	○	○	○

Others - Please explain

16. What are the clinical conditions in which POCUS is important?

	I disagree	I am not sure	I agree
Traumatic shock	○	○	○
Non traumatic shock	○	○	○
Acute abdomen	○	○	○
Dyspnea	○	○	○
CPR	○	○	○

Others - Please explain

17. Does your HEMS use standard protocols for POCUS?

- ○ Yes
- ○ No
- ○ Not sure

18. If the answer in the previous question was **YES** – What protocols are used?

- ☐ (p)FAST - (pre-hospital) focused assessment with sonography for trauma
- ☐ eFAST - extended focused assessment with sonography for trauma
- ☐ FATE - focus assessed transthoracic echo
- ☐ RUSH protocol for shock (Rapid Ultrasound in Shock and Hypotension)
- ☐ Other, please explain

POCUS in European HEMS

Point of care ultrasound (POCUS) in European Helicopter Emergency Medical Services.

4. Last but not least.

19. How often has POCUS being used in the last 1000 patients of your HEMS organization?

- ○ < 10
- ○ 10 - 50
- ○ 51 - 100
- ○ 101 - 200
- ○ 201 - 300
- ○ > 300
- ○ I do not know

20. How are the POCUS findings recorded in your HEMS?

- ☐ Video clip
- ☐ Electronic data base
- ☐ Mission protocol / Patient Record Form
- ☐ Not recorded, if not relevant
- ☐ Not recorded at all

21. What POCUS device do you use?

22. Are you pleased with this device or do you have remarks with respect to its use?

- Pleased
- Not pleased
- Remarks

23. Is there a credentialing process for using POCUS in your HEMS?

- Yes
- No
- Not sure

24. If the answer to the previous question was YES - What are the requirements?

Didactic teaching……….…. Hours

Hands on training………….Hours

Number of documented cases…………. Cases

POCUS-Course of an expert association in your country (1-YES; 0=No)

Prev Done

Powered by
SurveyMonkey®
See how easy it is to create a survey .
